# Negligible Effect of Quercetin in the Pharmacokinetics of Sulfasalazine in Rats and Beagles: Metabolic Inactivation of the Interaction Potential of Quercetin with BCRP

**DOI:** 10.3390/pharmaceutics13121989

**Published:** 2021-11-23

**Authors:** Ju-Hee Oh, Dokeun Kim, Haejun Lee, Gyeonghee Kim, Taehoon Park, Min Chang Kim, Young-Joo Lee

**Affiliations:** Division of Biopharmaceutics, College of Pharmacy, Kyung Hee University, Seoul 02447, Korea; 5juhee@khu.ac.kr (J.-H.O.); dk_kim@khu.ac.kr (D.K.); cj_xellos2@khu.ac.kr (H.L.); gyeong@khu.ac.kr (G.K.); neofiend@khu.ac.kr (T.P.); mcmr1218@khu.ac.kr (M.C.K.)

**Keywords:** quercetin, sulfasalazine, breast cancer resistance protein, bioavailability, interaction

## Abstract

Breast cancer resistance protein (BCRP) mediates pharmacokinetic drug interactions. This study evaluated the potential of quercetin to inhibit and induce BCRP in vitro and in vivo. The inhibition of BCRP was investigated for quercetin and its metabolites using BCRP/mBcrp1-overexpressing MDCKII cells by flow cytometry. The induction of BCRP was investigated in LS174T cells using quantitative PCR. The expression of rat BCRP in rat small intestine, liver, and kidney was also measured after multiple administrations of quercetin in rats (50, 100, and 250 mg/kg, seven days). The in vivo pharmacokinetic changes of sulfasalazine following single or multiple administration of quercetin in rats and beagles were investigated. Although the induction effect of quercetin on BCRP was observed in vitro, the in vivo expression of rat BCRP was not changed by multiple quercetin administrations. Oral administration of quercetin did not affect the plasma concentration or pharmacokinetic parameters of sulfasalazine, regardless of dose and dosing period in either rats or beagles. In addition, the inhibitory effect of quercetin metabolites on BCRP/mBcrp1 was not observed. These results suggest that the in vivo drug interaction caused by quercetin via BCRP was negligible, and it may be related to the metabolic inactivation of quercetin for the inhibition of BCRP.

## 1. Introduction

Herbal components in a daily meal (unintended uptake) or dietary supplement/traditional medicine (intended uptake) can affect the pharmacokinetics of concomitant medicines, resulting in a change in the safety and efficacy of drugs [1,2]. For example, 2621 adverse events associated with dietary supplements, including 101 deaths, were reported to the FDA over five years (2005–2010).

Together with P-glycoprotein (P-gp), breast cancer resistance protein (BCRP) is an efflux transporter restricting oral absorption and enhancing excretion of many compounds, including antivirals, lipid-lowering agents, antibiotics, and calcium channel blockers [3,4]. Since several classes of natural products have shown potent BCRP inhibition, herb–drug interactions mediated by BCRP could be a concern in patients who are taking phytochemical supplements [5]. For example, curcumin is known to inhibit the efflux of sulfasalazine via BCRP; therefore, systemic exposure of orally administered sulfasalazine concomitant with curcumin at a dose of 2000 mg/day was increased by 3.2-fold [6].

Quercetin is the most abundant plant flavonoid in the human diet, including in onions, apples, cappers, berries, and red wine. The average uptake of quercetin from daily food is 5–100 mg/day irrespective of the country, and quercetin contributes up to 70% of the flavonol and flavone uptake regardless of gender [7,8]. Quercetin has been used as a health supplement as quercetin alone or combined with other chemicals such as bromelain, other citrus flavonoids, or vitamins in the expectation of antioxidative, anti-inflammatory, antiviral, and anticancer activity, and the prevention of cardiovascular disease [1,9]. The recommended dose of commercially available quercetin as a health supplement is relatively high (average: 970 mg/day; mode: 500 mg/day; minimum: 250 mg/day; maximum: 4000 mg/day) [10].

Drug interactions caused by quercetin concerning drug transporters can be fatal. For example, Wang et al. reported that two-thirds of pigs administered digoxin, a typical substrate of P-gp, died unexpectedly after co-administration with quercetin; a rapid increase in digoxin plasma concentration was also observed [11].

Quercetin is a substrate and inhibitor of BCRP [12,13,14]. Sesink et al. determined that quercetin is a BCRP substrate with high efficiency [15]. In addition, the accumulation of BCRP substrates, such as SN-38, in BCRP-overexpressed cells was increased by quercetin; *K_i_* for SN-38 was 0.28 μM [16]. However, despite the potential of quercetin to cause herb–drug interactions through BCRP, in vivo research demonstrating this topic is hardly available except for the recently published study by Song et al. that showed unclear evidence of interaction by quercetin in vivo followed by oral administration at a dose of 10 mg/kg in rats [17].

This study investigated the pharmacokinetic interactions of quercetin with BCRP in vitro and in vivo concerning inhibition and induction. The pharmacokinetic interaction was investigated thoroughly at various doses and dosing days in rats and beagle dogs. In addition, the modulation of BCRP expression was investigated in vitro and in vivo. Finally, the potential for metabolic inactivation of the interaction was suggested.

## 2. Materials and Methods

### 2.1. Materials

Quercetin dihydrate, sulfasalazine, diclofenac, rhodamine123, verapamil, Ko143, and estrone were purchased from Sigma-Aldrich (St. Louis, MO, USA). Pheophorbide A was purchased from Frontier Scientific (Logan, UT, USA). A quercetin capsule commercially available from Solaray^®^ (Park City, UT, USA) was used for administration to beagle dogs. Unless indicated otherwise, all other chemicals were of analytical or HPLC grade, as appropriate. The LS174T cell line was obtained from the Korean Cell Line Bank (Seoul, Korea). Human P-gp, BCRP, and mouse Bcrp1-expressed MDCKII cells, as well as their control cells, were kindly gifted by Dr. Borst and Schinkel’s group [18,19,20].

### 2.2. Experimental Animal

Male Sprague-Dawley rats (220–330 g) were purchased from Samtako, Inc. (Osan, Korea) and were housed four per individual ventilated cage and given access to water ad libitum. Male beagle dogs weighing 12–16 kg were purchased from Orient Bio Inc. (Seongnam, Korea) and housed three per individual animal care room. They were allowed free access to water and fed 300 g of standard laboratory dog food once a day at 10:00 a.m.

All animals were kept in a controlled room at 25 ± 5 °C and 12 h light/dark cycles. All experiments were conducted according to protocols approved by the Committee on the Care and Use of Laboratory Animals of Kyung Hee University.

### 2.3. RNA Extraction and Quantitative PCR

The rats were orally administered 50, 100, and 250 mg/kg of quercetin suspended in 0.5% Na-carboxymethylcellulose orally for seven consecutive days. On the 8th day, the proximal 20 cm of the small intestine was extirpated following intraperitoneal injection of Zoletil^®^ (25 mg/kg of zolazepam and 25 mg/kg of tiletamine). Total RNA was extracted from the scraped intestinal mucosa using RNAiso (Takara, Tokyo, Japan) according to the manufacturer’s protocol. Subsequently, mRNA was reverse transcribed to cDNA using the Takara RNA PCR Kit (AMV ver. 3.0) and a Takara PCR Thermal Cycler Dice TP650. Quantitative PCR was performed in a LightCycler 1.5 (Roche Diagnostics, Mannheim, Germany). Thermocycling for each reaction was performed in a final volume of 20 μL reaction mixture using SYBR Green Premix Ex Taq (Takara Bio Inc., Otsu, Japan) and the primers listed in Table 1. The mRNA level was estimated by relative quantification to 18s RNA.

### 2.4. Cell Culture

P-gp, BCRP, and mouse Bcrp1-overexpressed MDCKII cells were subcultured in Dulbecco’s Modified Eagle Medium containing 100 units/mL penicillin, 100 μg/mL streptomycin, and 10% FBS and maintained at 37 °C in a humidified 5% CO_2_ atmosphere. LS174T cells were subcultured in the same way, except for the use of the RPMI1640 medium.

### 2.5. Changes in mRNA Levels of BCRP Expressed in LS174T Cells and Rat BCRP Expressed in the Liver, Kidney, and Small Intestine of Rats following Exposure to Quercetin

BCRP/rat BCRP mRNA was quantified in the cells and tissues of rats according to a previous report [24]. Briefly, LS174T cells were seeded at a density of 2 × 10^5^ cells/mL, followed by incubation in 5, 10, and 50 μM quercetin in 0.5% DMSO for 48 h with replacement every 24 h. Total RNA was extracted using RNAiso (Takara, Tokyo, Japan) and reverse transcribed to cDNA using the Takara RNA PCR™ Kit (AMV ver. 3.0) and Takara PCR Thermal Cycler Dice TP650. Quantitative PCR was performed using the LightCycler 1.5 (Roche Diagnostics, Mannheim, Germany). The mRNA levels of BCRP were calculated by relative quantification using the mRNA levels of glyceraldehyde-3-phosphate dehydrogenase.

Male SD rats were administered quercetin suspended in 0.5% sodium carboxymethyl cellulose at doses of 50, 100, and 250 mg/kg for seven consecutive days and liver, kidney, and small intestinal tissues were excised on the 8th day. RNA extraction and quantitative PCR were performed as described above. The mRNA level of Bcrp was calculated by relative quantification with 18s RNA.

### 2.6. Metabolic Degradation of Quercetin in Isolated Rat Hepatocytes

Freshly isolated rat hepatocytes were prepared according to the in situ collagenase perfusion method [25,26] and suspended in Krebs–Henseleit buffer (pH 7.4). The suspension of cells (0.5 × 10^6^ cells/0.5 mL) was incubated with quercetin (final concentration: 1 μM) in a 37 °C water shaking bath. After 20 min, the hepatocyte suspension was moved on ice and filtered using a 0.2 μM syringe filter and the filtrate was used as a mixture of quercetin metabolites. As the absolute amount of mixture of quercetin metabolites was unavailable, the amount of quercetin metabolites was considered the equivalent amount of quercetin used for initial incubation. The half-life was obtained by plotting the incubation time against the logarithmic concentration of quercetin.

### 2.7. Inhibitory Effect of Quercetin and Its Metabolites on BCRP, mBcrp1, and P-gp Using Flow Cytometry

The inhibitory effects of quercetin and its metabolites on BCRP, mBcrp1, and P-gp using flow cytometry were assessed as previously described [27] with minor modifications. Suspensions of BCRP, mBcrp1, and P-gp-overexpressing MDCKII cells were used for flow cytometry data collected on Guava EasyCyte (Millipore, MA, USA). The cellular accumulation of pheophorbide a, a selective substrate for BCRP and mBcrp1, and rhodamine 123, a selective substrate for P-gp, with or without quercetin (1 μM) or quercetin metabolites (1 μM equivalent) was evaluated [28]. The concentrations of pheophorbide a and rhodamine 123 were 10 μM and 1 μM, respectively. Verapamil (100 μM) and ko143 (10 μM) were used as positive control inhibitors of P-gp and BCRP, respectively. The uptake of all fluorescent substrates was evaluated using the peak fluorescence intensity [17].

### 2.8. Pharmacokinetic Study of Sulfasalazine in Rats and Beagles

The pharmacokinetic study of sulfasalazine in rats was designed to evaluate the effects of single-and multiple-dose quercetin. For the single-dose study, sulfasalazine (20 mg/kg) was administered orally with the vehicle for quercetin, quercetin (100 mg/kg), or estrone (100 mg/kg). Estrone was used as a positive control for BCRP inhibition [29]. For the multiple-dose-treated group, 100 mg/kg of quercetin or vehicle was administered orally for 7 consecutive days, and 20 mg/kg of sulfasalazine was administered to both groups on the 8th day. Three hundred microliters of blood were collected at 0 (pre-dose), 2, 10, 30, 60, 120, 180, 240, 360, 540, 720, and 1440 min and centrifuged at 16,850× *g* for 3 min to prepare plasma samples.

A 2 × 2 crossover design was used to evaluate the effect of co-administration of quercetin in beagles. Six beagle dogs were divided into two groups and administered 50 mg/kg of sulfasalazine in the gelatin capsule following an oral dose of 1 g/head or empty gelatin capsule. After a washout period of 1 week, they were titrated onto the alternative treatment (with quercetin or an empty gelatin capsule). For the multiple-dose study, an empty gelatin capsule or 1000 mg/head of quercetin mixed with food was administered to the control or treated group at 10:00 a.m. for seven consecutive days. On the 8th day, beagles were dosed with 50 mg/kg of sulfasalazine filled in a gelatin capsule orally. In addition, if quercetin were to cause a change in BCRP function such as inhibition or induction, it was thought that there would be a recovery of BCRP function with the interruption of quercetin dosing. To examine this, a recovery phase experiment was conducted. After a week-long washout period, sulfasalazine was administered again with or without a single dose of quercetin (1 g/head), and the pharmacokinetic profile was compared. Blood sampling for the pharmacokinetic study in beagle dogs was performed as follows: One milliliter of blood was collected from the cephalic vein of the foreleg at 0 (pre-dose), 2, 10, 30, 60, 120, 180, 240, 360, 540, 720, and 1440 min after administration. Three hours after drug administration, the beagle dogs were released from the animal jacket and provided food. Thereafter, they were kept in the care room with ad libitum access to water until blood sampling was completed.

Pharmacokinetic parameters of sulfasalazine were determined using the model-independent method using Excel^TM^. The AUC from zero to the last measurable point (AUC_last_) was used to evaluate the amount of absorption. The observed peak concentration (C_max_) and time to reach C_max_ (T_max_) were used to evaluate the amount of and the rate of absorption, respectively. Considering the double peak phenomenon observed for sulfasalazine, the first observed peak concentration was considered to be C_max_ [30].

### 2.9. LC-MS/MS Analysis

The plasma concentration of sulfasalazine was analyzed according to a previous report, with minor modifications [31,32]. Plasma (20 μL) was vortex-mixed with 200 μL of acetonitrile containing 50 ng/mL diclofenac for 5 min and centrifuged at 16,850× *g* at 18 °C for 3 min. Ten microliters of supernatant were injected into *Sepax*GP-C18 (2.1 × 50 mm, 3 μM, 120 Å, Sepax Technologies, Inc., Newark, DE, USA) connected to an Agilent 1200 series (Agilent, Santa Clara, CA, USA). The temperature of the samples in the autosampler was maintained at 4 °C. The mobile phase consisted of 0.16 mM ammonium formate (solvent A) and acetonitrile (solvent B). The gradient eluent was as follows, with a flow rate of 0.3 mL/min: Solvent B was held at 30% for 0.1 min, linearly ramped from 30% to 95% in 1.4 min, held at 95% for 1.5 min, and then immediately brought back down to 30% for re-equilibration. A Waters Quattro micro™ API mass spectrometer (Waters, Milford, MA, USA) equipped with an electrospray ionization source was used as a detector. The analysis was performed in negative ion ESI mode with a capillary voltage of 3.00 kV, cone voltage of 45 V, source temperature of 120 °C, and desolvation temperature of 800 °C. A cone gas flow of 50 L/h and desolvation gas flow of 800 h/L were used. Nitrogen was used as the nebulizing and desolvation gas, and argon was used as the collision gas. Multiple reaction monitoring analysis was performed with the transitions *m/z* 397 → 197.1 and *m/z* 294 → 250 for sulfasalazine and diclofenac, respectively. All data were collected and processed using MassLynx 4.1 software (Water, Milford, MA, USA). The range of standard curve was 6.25~2000 ng/mL. The limit of quantification was 6.25 ng/mL. If a sample concentration exceeded the highest calibration standard, the sample was diluted with blank plasma and reassayed. The accuracy and precision values of the quality control samples were within the acceptable limits (<15%).

The analysis of quercetin in isolated hepatocyte suspensions was performed according to a previous report [33].

### 2.10. Statistical Analysis

Statistical differences between the control and treated groups were determined using an unpaired *t*-test for rats and beagles. For single-administered beagles, a paired *t*-test was used. For mRNA expression, one-way ANOVA was used, followed by post-hoc LSD. Statistical significance was set at *p* < 0.05. Statistical analysis was performed with SPSS, version 21 (SPSS Inc., Chicago, IL, USA).

## 3. Results

### 3.1. Modulation of the Expression Level of BCRP by Administration of Quercetin

**Figure 1:**
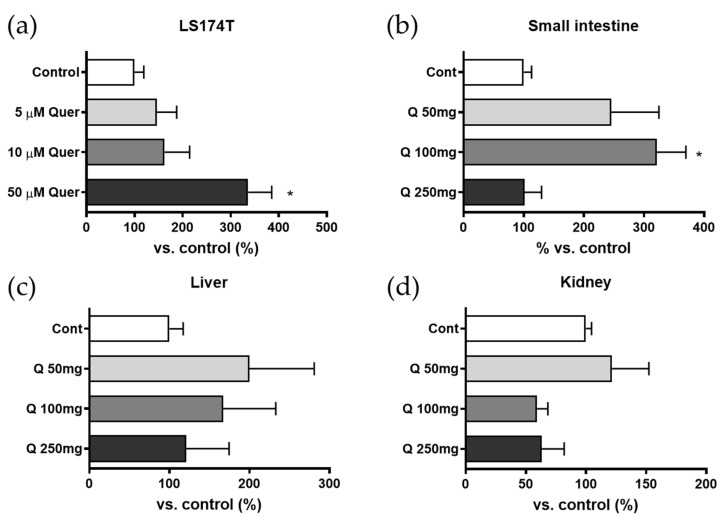
mRNA expression of BCRP (**a**) in LS174T cells and rat BCRP expression (**b**–**d**) in rat small intestine, liver, and kidney. LS174T cells were incubated for 48 h with quercetin (5 μM, 10 μM, and 50 μM), and rats were administered 50, 100, and 250 mg/kg of quercetin for seven consecutive days. Each value represents mean ± SE for the percentage compared to the vehicle-treated group (*n* = 3, * *p* < 0.05).

Figure 1 shows the mRNA expression of BCRP in the LS174T cells, small intestine, liver, and kidney of rats. The mRNA expression level of BCRP in LS174T cells increased with the concentration of pretreated quercetin and showed a statistical difference for the 50 μM-treated group. Long-term oral exposure to quercetin in vivo (50 and 100 mg/kg for seven consecutive days) increased the mRNA expression of BCRP in the small intestine, and a statistically significant increase in the expression of BCRP was observed in the group administered 100 mg/kg. The 250 mg/kg quercetin treatment group did not show any difference in the expression level of BCRP. The mRNA expression of BCRP in the liver and kidney did not show clear modulation because of the relatively large deviations in expression levels.

### 3.2. Negligible In Vivo Pharmacokinetic Change of Sulfasalazine Followed by Quercetin Administration

**Figure 2:**
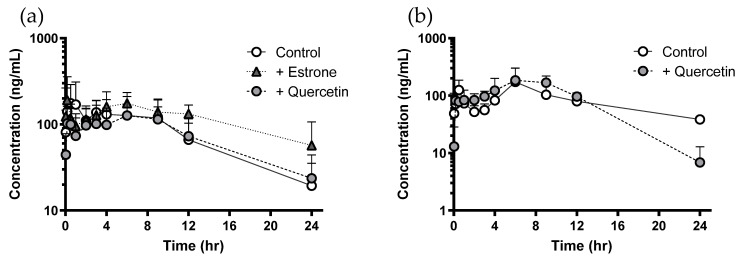
Plasma concentration of sulfasalazine following single (**a**) and multiple administration (**b**) of quercetin in rats. Data were shown as mean ± SD ((**a**) *n* = 6, (**b**) *n* = 3). Open and closed circles represent the control and quercetin-treated group, respectively, and triangles represent the estrone-treated group.

Figure 2 represent the plasma concentration profile of sulfasalazine with or without a single dose of quercetin (100 mg/kg) and multiple doses of quercetin for seven days in rats. Despite well-documented in vitro evidence to support the possibility of BCRP-mediated interaction caused by quercetin, administration of quercetin (single or multiple) did not result in any significant difference in the plasma concentration of sulfasalazine. Only the plasma concentrations of sulfasalazine at 24 h in the multiple-dose group were significantly lower than those in the control group (Figure 2b). However, co-administration of estrone (positive control of the BCRP inhibitor) increased the plasma concentration of sulfasalazine. The pharmacokinetic parameters are summarized in Table 2. No significant change in the pharmacokinetics of sulfasalazine was observed after single or multiple administrations of quercetin in rats. However, there was a significant increase in the AUC of the positive control group treated with estrone.

**Figure 3:**
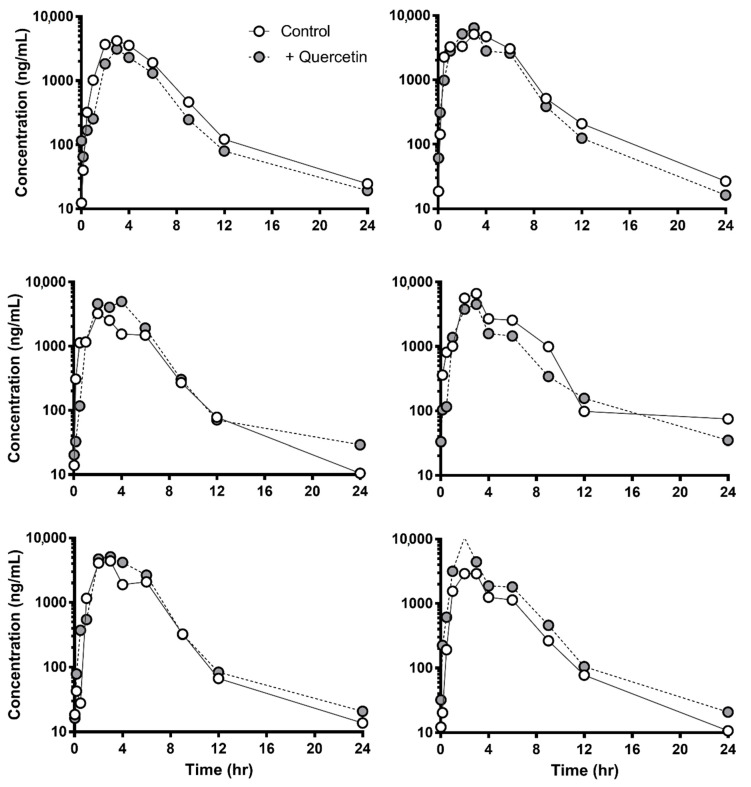
Plasma concentration of sulfasalazine with or without single administration of quercetin (1 g/head) in each beagle dog (*n* = 6). Open and closed circles represent the control and quercetin-treated group, respectively.

**Figure 4:**
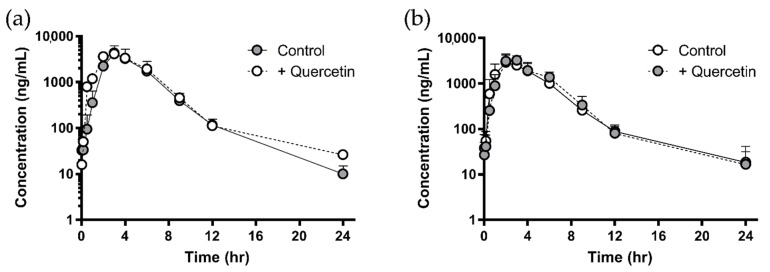
Plasma concentration of sulfasalazine following multiple administration (**a**) of quercetin and recovery phase (**b**) in beagle dogs. Data are shown as mean ± SD (*n* = 3). Open and closed circles represent the control and quercetin-treated group, respectively.

Figure 3 and Figure 4 show the plasma concentration profile of sulfasalazine with or without a single dose (1 g/head) or multiple doses (for seven days) of quercetin in dogs. Similar to the results in rats, administration of quercetin did not cause any significant difference in the plasma profile or pharmacokinetic parameters of sulfasalazine (Table 3).

### 3.3. Loss of BCRP Inhibitory Activity by Metabolic Degradation of Quercetin

**Figure 5:**
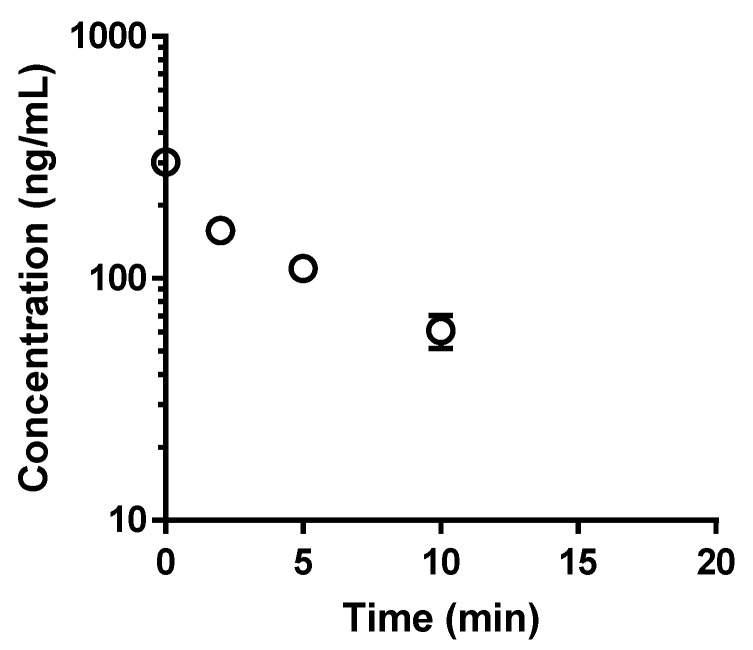
The disappearance of quercetin in isolated rat hepatocytes. Hepatocytes were incubated at 10^6^ cells/mL in Krebs–Henseleit buffer, pH 7.4, as described in the Materials and Methods section. Results are expressed as the means ± SD of three experiments.

The metabolic instability of quercetin is shown in Figure 5. Based on previous studies, quercetin rapidly degraded in isolated rat hepatocytes with a half-life of 4.6 min. Because over 95% of quercetin in the suspension of hepatocyte may exist as its metabolites after 20 min, the filtered incubation solution was used as a mixture of quercetin metabolites.

To evaluate the inhibitory activity of quercetin metabolites, a FACS flow cytometry study using BCRP- and mBcrp1-overexpressing MDCKII cells was performed for quercetin and metabolites of quercetin that were generated by isolated rat hepatocytes.

**Figure 6:**
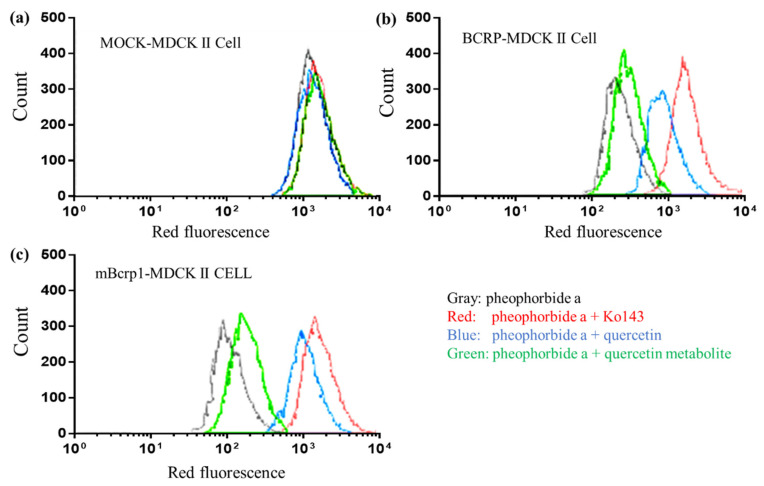
Representative histogram of pheophorbide a accumulation in MOCK-MDCKII cells (**a**), BCRP-overexpressed MDCKII cells (**b**), and mBcrp1-overexpressed MDCKII cells (**c**). Fluorescence of pheophorbide a in MOCK-MDCKII cells was not affected by Ko143, quercetin, or quercetin metabolites (**a**). However, quercetin and Ko143 increased the accumulation of pheophorbide a in BCRP-overexpressed MDCKII cells (**b**) and mBcrp1-overexpressed MDCKII cells (**c**) by inhibition of BCRP function, whereas the inhibitory effect of quercetin metabolites was not observed.

As expected, the fluorescence intensity of pheophorbide a, a well-known BCRP-specific marker, was lower in BCRP and mBcrp1-overexpressing cells than that in MOCK-MDCKII cells (Figure 6). In addition, quercetin, quercetin metabolites, and the BCRP inhibitor Ko143 did not lead to any clear shift in the peak fluorescence intensity of pheophorbide a in MOCK-MDCKII cells (<0.25-fold shift for all groups).

Figure 6 shows that quercetin and the BCRP inhibitor Ko143 could inhibit the function of BCRP and mBcrp1. In the Ko143 co-treated group, the peak shift in fluorescence intensity was 7.5-fold in BCRP-MDCKII cells and 15.7-fold in mBcrp1-MDCKII cells. Quercetin co-treatment showed a similar shift pattern (4.3-fold in BCRP-MDCKII cells and 10.5-fold in mBcrp1-MDCKII cells). Interestingly, equivalent molar amounts of quercetin metabolites did not show clear inhibition of BCRP and mBcrp1, where the fold shift was only 30.8% and 16.6% of that induced by quercetin in BCRP-MDCKII cells and mBcrp1-MDCKII cells, respectively.

**Figure 7:**
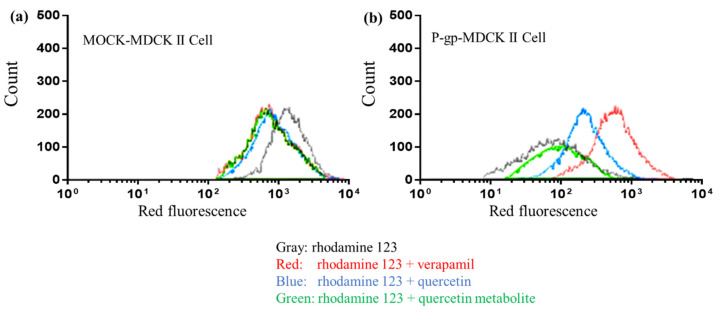
Representative histogram of rhodamine 123 accumulation in MOCK-MDCKII cells (**a**) and P-gp-overexpressed MDCKII cells (**b**). Fluorescence of rhodamine 123 accumulation in MOCK-MDCKII cells was not affected by verapamil, quercetin, or quercetin metabolites (**a**). However, quercetin and verapamil increased the accumulation of rhodamine 123 in P-gp-overexpressed MDCKII cells, whereas quercetin metabolites did not show the inhibitory effect (**b**).

These results were also reproduced in the P-gp-overexpressing cells. Quercetin and verapamil, the positive control of the P-gp inhibitor, inhibited the function of P-gp, although equivalent molar metabolites of quercetin did not (Figure 7).

## 4. Discussion

Although BCRP recognizes various drugs as a substrate and its importance in intestinal absorption has grown, information on the absorptive interaction between BCRP and quercetin in vivo has not yet been fully elucidated. Given in vitro reports in the literature that showed possible interaction caused by quercetin via BCRP and the upregulation of BCRP mRNA expression in LS174T cells in our results, we postulate that single and multiple doses of quercetin could affect the bioavailability of drugs recognized by BCRP. For inhibition studies of BCRP, substrate selection is critical because its substrates often overlap with P-gp; therefore, data interpretation could be confused. Fortunately, Zaher et al. revealed that sulfasalazine has a higher affinity for BCRP (with a *K_m_* value below 1 μM) than P-gp, and the AUC ratio of knockout mice to wild-type mice was 55.5-fold higher in Bcrp1^−/−^ knockout mice than in Mdr1a^-/-^ knockout mice [32,34]. Therefore, we investigated the effect of quercetin on the intestinal absorption of sulfasalazine through BCRP.

Because our mRNA data in LS174T cells and in vivo suggested the possibility of the induction of BCRP by chronic administration of quercetin, we evaluated the effect of quercetin on the pharmacokinetics of sulfasalazine after multiple doses of quercetin in addition to a single dose of quercetin.

We administered quercetin at a dose of 100 mg/kg for rats and 1 g/head for dogs because the daily dose of quercetin is in the range of 250 to 4000 mg. The human equivalent dose, considering body surface area, for a rat dose of 100 mg/kg corresponds to 1129 mg/70 kg. Thus, the dose we used in the experiment is thought to reflect the clinical situation.

Although some evidence suggests that quercetin can change the bioavailability of drugs whose penetration through the intestinal epithelium is limited by BCRP, quercetin did not affect the intestinal absorption of sulfasalazine following single and multiple administrations in rats. Recently, Song et al. also reported that an oral dose of quercetin at a dose of 10 mg/kg did not change the pharmacokinetics of co-administered sulfasalazine [17]. Only the plasma concentrations of sulfasalazine at 24 h in the multiple-dose group were significantly lower than those in the control group (Figure 2b). It was difficult to consider, however, that inhibition of BCRP-mediated absorption of sulfasalazine by quercetin resulted in the decreased plasma concentration at 24 h. In addition, similar results were not observed in the dog study (Figure 4a).

Concerning the discrepancy between in vitro BCRP inhibition results and in vivo pharmacokinetic results, several hypotheses, including species differences or the feasibility of sulfasalazine as a maker, can be argued. However, Figure 6 clearly shows that both BCRP and mBcrp1 were inhibited by quercetin. In addition, a lack of interaction between quercetin and sulfasalazine was observed in rats and beagles. Thus, inter-species differences affecting the impact of quercetin on the absorption of sulfasalazine seem to be negligible. Another possibility, that sulfasalazine was not a good marker of oral absorption in the BCRP-mediated interaction study, was also excluded. BCRP expression increased along the intestine from the proximal to the distal region with maximal expression in the ileum and decreased in the colon for both mRNA and protein; protein expressed in the ileum was about 3–5-fold compared to that in the duodenum [35]. A considerable amount of sulfasalazine may remain in its parent form in the small intestinal tract because the bioavailability of sulfasalazine is very low, and metabolism barely occurs in the small intestine. Thus, the chances of interaction with BCRP might be high [36,37].

We considered other reasons to explain the negligible effect of quercetin on sulfasalazine absorption, regardless of species. We supposed that intestinal concentration of quercetin was too low to lead to altering the BCRP activity because of extensive metabolism. Crespy et al. reported that under 40% of the initial dose of quercetin existed as a parent form in the intestinal lumen after intestinal perfusion due to the extensive metabolism of quercetin [38]. Our metabolic stability study in isolated rat hepatocytes also showed the metabolic instability of quercetin. The critical point of this hypothesis is whether quercetin metabolites also inhibit the function of BCRP. Therefore, we tested the inhibitory effects of quercetin metabolites using FACS cytometric analysis. Quercetin has been reported to yield various metabolites, including quercetin-3-glucuronide and quercetin-3′-sulfate as a major form in rats and humans [39,40]. We used the remaining reaction mixture after incubation in isolated rat hepatocytes as an equivalent molar metabolite mixture of quercetin. As a result, we found that the metabolite mixture of quercetin did not inhibit the function of BCRP or mBcrp1. These results were supported by recent literature [41] that showed that the IC50 of quercetin-3-glucuronide (24.2 μM) and quercetin-3′-sulfate for BCRP (3.2 μM) were much higher than that of quercetin (0.13 μM). Considering that the metabolic process is a self-defense mechanism that includes the deactivation of the pharmacological activity of drugs, this result could be expected. In addition, gastric absorption of quercetin may also contribute to the lack of interaction for the intestinal absorption of sulfasalazine partially because approximately 38% of quercetin disappeared in the stomach (maybe absorbed) [42] and the remains (approximately a half-dose) could have been transferred to the small intestine, which is the site of interaction.

Another interesting point is that the metabolic degradation of quercetin negated its inhibitory effects on P-gp (Figure 6), whereas both quercetin and its metabolites inhibited CYP3A [43,44]. These results suggest a possible need to carefully review the inhibitory effect of quercetin on P-gp in published results. Since drugs transported by P-gp tend also to be metabolized by CYP3A [45], it remains possible that the in vivo interaction caused by quercetin results from the inhibition of CYP3A, not of P-gp.

## 5. Conclusions

In summary, this study demonstrated that quercetin did not affect the intestinal absorption of sulfasalazine through BCRP in rats and beagles, irrespective of the exposure period. This may be related to metabolic inactivation and low exposure of parent quercetin in intestinal cells resulting from metabolism.

## Figures and Tables

**Table 1:** Primers and real-time PCR conditions were used in quantification of mRNA expression in LS174T cells and rats.

Gene	Accession No.	Sequence (5′-3′)	Condition
r18s RNA [21]	M11188	FW- CGCCGCTAGAGGTGAAATTC RW- CCAGTCGGCATCGTTTATGG	95 °C—10 s, 95 °C—5 s, 61 °C—7 s, 72 °C—10 s
Rat BCRP [22]	NM_181381	FW- CAGCAGGTTACCACTGTGAG RW- TTCCCCTCTGTTTAACATTACA	95 °C—10 s, 95 °C—5 s, 56 °C—6 s, 72 °C—6s
Human BCRP [23]	NM_004827.2	FW- CAGGTCTGTTGGTCAATCTCACA RW- TCCATATCGTGGAATGCTGAAG	95 °C—10 s, 95 °C—5 s, 58 °C—15 s, 72 °C—15 s

**Table 2:** Pharmacokinetic parameters of sulfasalazine following single and multiple administration of quercetin in rats. Data are shown as mean ± SD (** *p* < 0.01).

	Single Administration (*n* = 6)			Multiple Administration (*n* = 3)	
	Control	+Quercetin	+Estrone	Control	+Quercetin
AUC_last_ (μg∙h/mL)	1.84 ± 0.557	1.65 ± 0.912	2.73 ± 0.301 **	1.87 ± 0.684	1.87 ± 0.367
C_max_ (ng/mL)	226 ± 130	126 ± 64	277 ± 118	141 ± 34	111 ± 10
T_max_ (h)	1.06 ± 1.47	0.506 ± 0.749	3.39 ± 3.61	0.389 ± 0.192	1.72 ± 1.44

**Table 3:** Pharmacokinetic parameters of sulfasalazine following single and multiple administration of quercetin in beagle dogs. Data are shown as mean ± SD.

	Single Administration (*n* = 6)		Multiple Administration (*n* = 3)			
			7-Day Treatment		Recovery Phase	
	Control	+Quercetin	Control	+Quercetin	Control	+Quercetin
AUC_last_ (μg∙h/mL)	21.0 ± 6.80	21.4 ± 5.63	18.1 ± 7.85	21.0 ± 7.20	13.7 ± 6.34	15.1 ± 3.15
C_max_ (μg/mL)	4.41 ± 1.35	4.83 ± 1.19	4.40 ± 1.79	4.23 ± 1.09	3.19 ± 1.24	3.64 ± 0.916
T_max_ (h)	2.83 ± 0.408	3.20 ± 0.447	3.33 ± 0.577	3.00 ± 1.00	2.00 ± 1.00	2.67 ± 0.577

## References

[1] Tarirai C., Viljoen A.M., Hamman J.H. (2010). Herb–drug pharmacokinetic interactions reviewed. Expert Opin. Drug Metab. Toxicol..

[2] De Smet P.A.G.M. (2007). Clinical risk management of herb-drug interactions. Br. J. Clin. Pharmacol..

[3] Pangeni R., Kang S., Jha S.K., Subedi L., Park J.W. (2021). Intestinal membrane transporter-mediated approaches to improve oral drug delivery. J. Pharm. Investig..

[4] Huls M., Brown C., Windass A., Sayer R., Heuvel J.V.D., Heemskerk S., Russel F., Masereeuw R. (2008). The breast cancer resistance protein transporter ABCG2 is expressed in the human kidney proximal tubule apical membrane. Kidney Int..

[5] Robey R.W., To K.K., Polgar O., Dohse M., Fetsch P., Dean M., Bates S.E. (2009). ABCG2: A perspective. Adv. Drug Deliv. Rev..

[6] Kusuhara H., Furuie H., Inano A., Sunagawa A., Yamada S., Wu C., Fukizawa S., Morimoto N., Ieiri I., Morishita M. (2012). Pharmacokinetic interaction study of sulfasalazine in healthy subjects and the impact of curcumin as an in vivo inhibitor of bcrp. Br. J. Pharmacol..

[7] Sampson L., Rimm E., Hollman P.C., de Vries J.H., Katan M.B. (2002). Flavonol and Flavone Intakes in US Health Professionals. J. Am. Diet. Assoc..

[8] Harwood M., Danielewska-Nikiel B., Borzelleca J., Flamm G., Williams G., Lines T. (2007). A critical review of the data related to the safety of quercetin and lack of evidence of in vivo toxicity, including lack of genotoxic/carcinogenic properties. Food Chem. Toxicol..

[9] Bors W., Heller W., Michel C., Saran M. (1990). [36] Flavonoids as antioxidants: Determination of radical-scavenging efficiencies. Methods Enzymol..

[10] Vida R.G., Fittler A., Somogyi-Végh A., Poór M. (2019). Dietary quercetin supplements: Assessment of online product informations and quantitation of quercetin in the products by high-performance liquid chromatography. Phytother. Res..

[11] Wang Y.-H., Chao P.-D.L., Hsiu S.-L., Wen K.-C., Hou Y.-C. (2004). Lethal quercetin-digoxin interaction in pigs. Life Sci..

[12] Brand W., Schutte M.E., Williamson G., van Zanden J.J., Cnubben N.H., Groten J.P., van Bladeren P.J., Rietjens I. (2006). Flavonoid-mediated inhibition of intestinal ABC transporters may affect the oral bioavailability of drugs, food-borne toxic compounds and bioactive ingredients. Biomed. Pharmacother..

[13] Cooray H.C., Janvilisri T., van Veen H.W., Hladky S.B., Barrand M.A. (2004). Interaction of the breast cancer resistance protein with plant polyphenols. Biochem. Biophys. Res. Commun..

[14] Zhang S., Yang X., Morris M.E. (2004). Flavonoids Are Inhibitors of Breast Cancer Resistance Protein (ABCG2)-Mediated Transport. Mol. Pharmacol..

[15] Sesink A.L., Arts I.C., de Boer V.C., Breedveld P., Schellens J.H., Hollman P.C., Russel F.G. (2005). Breast cancer resistance protein (bcrp1/abcg2) limits net intestinal uptake of quercetin in rats by facilitating apical efflux of glucuronides. Mol. Pharmacol..

[16] Yoshikawa M., Ikegami Y., Sano K., Yoshida H., Mitomo H., Sawada S., Ishikawa T. (2004). Transport of SN-38 by the wild type of human ABC transporter ABCG2 and its inhibition by quercetin, a natural flavonoid. J. Exp. Ther. Oncol..

[17] Song Y.-K., Yoon J.-H., Woo J.K., Kang J.-H., Lee K.-R., Oh S.H., Chung S.-J., Maeng H.-J. (2020). Quercetin Is a Flavonoid Breast Cancer Resistance Protein Inhibitor with an Impact on the Oral Pharmacokinetics of Sulfasalazine in Rats. Pharmaceutics.

[18] Bakos É., Evers R., Szakacs G., Tusnády G.E., Welker E., Szabó K., de Haas M., van Deemter L., Borst P., Váradi A. (1998). Functional Multidrug Resistance Protein (MRP1) Lacking the N-terminal Transmembrane Domain. J. Biol. Chem..

[19] Pavek P., Merino G., Wagenaar E., Bolscher E., Novotna M., Jonker J.W., Schinkel A.H. (2005). Human Breast Cancer Resistance Protein: Interactions with Steroid Drugs, Hormones, the Dietary Carcinogen 2-Amino-1-methyl-6-phenylimidazo(4,5-b)pyridine, and Transport of Cimetidine. J. Pharmacol. Exp. Ther..

[20] Jonker J., Smit J.W., Brinkhuis R.F., Maliepaard M., Beijnen J.H., Schellens J.H.M., Schinkel A.H. (2000). Role of Breast Cancer Resistance Protein in the Bioavailability and Fetal Penetration of Topotecan. J. Natl. Cancer Inst..

[21] Uno S., Uraki M., Ito A., Shinozaki Y., Yamada A., Kawase A., Iwaki M. (2009). Changes in mRNA expression of ABC and SLC transporters in liver and intestines of the adjuvant-induced arthritis rat. Biopharm. Drug Dispos..

[22] Declèves X., Bihorel S., Debray M., Yousif S., Camenisch G., Scherrmann J.-M. (2008). ABC transporters and the accumulation of imatinib and its active metabolite CGP74588 in rat C6 glioma cells. Pharmacol. Res..

[23] Brand W., Van Der Wel P.A., Rein M.J., Barron D., Williamson G., Bladeren P.J., Rietjens I.M. (2008). Metabolism and Transport of the Citrus Flavonoid Hesperetin in Caco-2 Cell Monolayers. Drug Metab. Dispos..

[24] Oh J.-H., Lee J.H., Lee Y.-J. (2019). Evaluation of the Mrp2-mediated flavonoid-drug interaction potential of quercetin in rats and in vitro models. Asian J. Pharm. Sci..

[25] Berry M.N., Friend D.S. (1969). High-yield preparation of isolated rat liver parenchymal cells: A biochemical and fine structural study. J. Cell Biol..

[26] Shen L., Hillebrand A., Wang D.Q.-H., Liu M. (2012). Isolation and Primary Culture of Rat Hepatic Cells. J. Vis. Exp..

[27] Robey R., Honjo Y., van de Laar A., Miyake K., Regis J.T., Litman T., Bates S.E. (2001). A functional assay for detection of the mitoxantrone resistance protein, MXR (ABCG2). Biochim. Biophys. Acta Biomembr..

[28] Robey R.W., Steadman K., Polgar O., Morisaki K., Blayney M., Mistry P., Bates S.E. (2004). Pheophorbide a Is a Specific Probe for ABCG2 Function and Inhibition. Cancer Res..

[29] Tsukahara S., Imai Y., Sugimoto Y., Ueda K., Tsuruo T. (2003). Reversal of breast cancer resistance protein-mediated drug resistance by estrogen antagonists and agonists. Mol. Cancer Ther..

[30] Zamek-Gliszczynski M.J., Bedwell D.W., Bao J.Q., Higgins J.W. (2012). Characterization of SAGE Mdr1a (P-gp), Bcrp, and Mrp2 Knockout Rats Using Loperamide, Paclitaxel, Sulfasalazine, and Carboxydichlorofluorescein Pharmacokinetics. Drug Metab. Dispos..

[31] Urquhart B.L., Ware J.A., Tirona R.G., Ho R.H., Leake B.F., Schwarz U.I., Zaher H., Palandra J., Gregor J.C., Dresser G.K. (2008). Breast cancer resistance protein (ABCG2) and drug disposition: Intestinal expression, polymorphisms and sulfasalazine as an in vivo probe. Pharm. Genom..

[32] Zaher H., Khan A.A., Palandra J., Brayman T.G., Yu L., Ware J.A. (2006). Breast Cancer Resistance Protein (Bcrp/abcg2) Is a Major Determinant of Sulfasalazine Absorption and Elimination in the Mouse. Mol. Pharm..

[33] Park H.S., Oh J.H., Lee J., Lee Y.J. (2011). Minor effects of the Citrus flavonoids naringin, naringenin and quercetin, on the pharmacokinetics of doxorubicin in rats. Pharmazie.

[34] Jani M., Szabó P., Kis E., Molnár É., Glavinas H., Krajcsi P. (2009). Kinetic Characterization of Sulfasalazine Transport by Human ATP-Binding Cassette G2. Biol. Pharm. Bull..

[35] MacLean C., Moenning U., Reichel A., Fricker G. (2008). Closing the Gaps: A Full Scan of the Intestinal Expression of P-Glycoprotein, Breast Cancer Resistance Protein, and Multidrug Resistance-Associated Protein 2 in Male and Female Rats. Drug Metab. Dispos..

[36] Azadkhan A.K., Truelove S.C., Aronson J.K. (1982). The disposition and metabolism of sulphasalazine (salicylazosulphapyridine) in man. Br. J. Clin. Pharmacol..

[37] Chungi V.S., Dittert L.W., Shargel L. (1989). Pharmacokinetics of Sulfasalazine Metabolites in Rats Following Concomitant Oral Administration of Riboflavin. Pharm. Res..

[38] Crespy V., Morand C., Manach C., Besson C., Demigne C., Remesy C. (1999). Part of quercetin absorbed in the small intestine is conjugated and further secreted in the intestinal lumen. Am. J. Physiol. Content.

[39] Mullen W., Edwards C.A., Crozier A. (2006). Absorption, excretion and metabolite profiling of methyl-, glucuronyl-, glucosyl- and sulpho-conjugates of quercetin in human plasma and urine after ingestion of onions. Br. J. Nutr..

[40] Yeh S.-L., Lin Y.-C., Lin Y.-L., Li C.-C., Chuang C.-H. (2015). Comparing the metabolism of quercetin in rats, mice and gerbils. Eur. J. Nutr..

[41] Mohos V., Fliszár-Nyúl E., Ungvári O., Kuffa K., Needs P.W., Kroon P.A., Telbisz Á., Özvegy-Laczka C., Poór M. (2020). Inhibitory Effects of Quercetin and Its Main Methyl, Sulfate, and Glucuronic Acid Conjugates on Cytochrome P450 Enzymes, and on OATP, BCRP and MRP2 Transporters. Nutrients.

[42] Crespy V., Morand C., Besson C., Manach C., Demigne C., Remesy C. (2002). Quercetin, but not Its Glycosides, Is Absorbed from the Rat Stomach. J. Agric. Food Chem..

[43] Yang T., Liu Y., Huang X., Zhang R., Yang C., Zhou J., Zhang Y., Wan J., Shi S. (2018). Quercetin3obetadglucoside decreases the bioavailability of cyclosporin a through regulation of drug metabolizing enzymes, transporters and nuclear receptors in rats. Mol. Med. Rep..

[44] Östlund J., Žlábek V., Zamaratskaia G. (2017). In vitro inhibition of human CYP2E1 and CYP3A by quercetin and myricetin in hepatic microsomes is not gender dependent. Toxicology.

[45] Pal D., Mitra A.K. (2006). MDR- and CYP3A4-Mediated Drug–Drug Interactions. J. Neuroimmune Pharmacol..

